# When a portion becomes a norm: Exposure to a smaller vs. larger portion of food affects later food intake

**DOI:** 10.1016/j.foodqual.2019.02.013

**Published:** 2019-07

**Authors:** Eric Robinson, Jodie Henderson, Gregory S. Keenan, Inge Kersbergen

**Affiliations:** a Institute of Psychology, Health & Society, University of Liverpool, L69 7ZA, UK; b School of Health and Related Research, University of Sheffield, S1 4DA, UK

**Keywords:** BMI, Body mass index, DEBQ, Dutch eating behaviour questionnaire, Portion size, Food environment, Obesity, Social norms

## Abstract

**Background:**

Portion sizes in the food environment may communicate information about what constitutes a ‘normal’ amount of food to eat. Here we examined whether mere visual exposure to a smaller vs. larger portion size of snack food affects perceptions of how much a ‘normal’ sized portion is and how much people choose to eat of that food in future.

**Methods:**

Under the guise of a study on taste preference and personality, 104 female participants were randomly allocated to be exposed to either a smaller or larger portion size of snack food. Twenty-four hours later participants freely selected a portion of the snack food to consume and reported on their perception of what constituted a normal sized portion of the snack food.

**Results:**

Participants that were exposed to a smaller, as opposed to larger portion size subsequently believed that a normal portion of the snack food was smaller in size. Exposure to the smaller as opposed to the larger portion size also resulted in participants consuming less snack food the next day.

**Conclusions:**

Environmental exposure to smaller, as opposed to larger portion sizes of food may change perceptions of what constitutes a normal amount of food to eat and affect the amount of food people choose to eat in future.

## Highlights

• Portion size affects acute food intake.
• Portion size may also affect consumer portion size preferences.
• We tested whether smaller portions ‘normalize’.
• Consumer perceptions changed after seeing a smaller portion.
• Consumers ate less in future after seeing a smaller portion.

## 1 Introduction

Changes to the food environment have been identified as a potential contributor to the obesity crisis. In particular, the portion sizes of many food products have increased over time and there is now reliable evidence that larger food portion sizes increase concurrent food intake (Livingstone and Pourshahidi, 2014, Zlatevska et al., 2014). One of the reasons that portion size is thought to influence food intake is because portion sizes may communicate information about what a ‘normal’ amount of food to eat is (Haynes et al., 2019, Herman et al., 2015, Versluis and Papies, 2016).

Given the consistent effect that portion size has on food intake, there is a growing public health interest in the potential impact of reducing food portion sizes (Marteau, Hollands, Shemilt, & Jebb, 2015). A number of studies suggest that large portion sizes have a prolonged effect on energy intake and that reducing portion size decreases energy intake (Lewis et al., 2015, Rolls et al., 2006, Rolls et al., 2007). Yet, other potential downstream consequences of reducing portion sizes have received less attention. A recent study (Robinson & Kersbergen, 2018) showed that after eating a smaller, as opposed to larger portion size of a main meal food, participants tended to select a smaller portion of that food to eat one day and one week later. These findings were explained by participants having adjusted their perception of what constituted a normal portion size of the main meal in response to the portion size they had earlier eaten, which is in line with the notion that portion size can communicate normative information (Haynes et al., 2019, Kerameas et al., 2015, Versluis and Papies, 2016). However, a different interpretation that does not rely on portion size communicating normative information and therefore cannot be ruled out in these studies is that after consuming a smaller portion size, consumers learn about the post-ingestive consequences of eating that portion size (Gibson & Brunstrom, 2007) and adjust their preferences accordingly (e.g. a smaller portion is sufficiently satiating). Given the limited research to date, it is unclear whether exposure to smaller vs. larger portion sizes reliably affects later dietary behaviour and whether this is because portion size communicates normative information.

In the present study we examined whether mere visual exposure to a smaller vs. larger portion size of a snack food would affect food intake twenty-four hours later, in the absence of having initially eaten a smaller vs. larger portion of that food. Based on the premise that portion size may communicate information about what a normal amount of food to eat is, we predicted that being exposed to a smaller as opposed to larger portion size of a snack food would result in participants later believing a ‘normal’ portion size of the snack food to be smaller and freely choosing to eat less of the snack food in future.

## 2 Method

### 2.1 Overview

Under the guise of a study on ‘taste preference and personality’ participants attended two laboratory sessions in a between-subjects experiment. In session one participants were randomly allocated to be exposed to a smaller vs. larger portion size of snack food, before returning one day later for a second session. In the second session participants selected a portion of the snack food to consume and reported on their perception of what constituted a normal portion size of the snack food.

### 2.2 Sample

As the influence portion size has on food intake has been shown to vary according to gender (Zlatevska et al., 2014), we minimized heterogeneity by recruiting females only. We based our sample size (approximately 50 participants per condition) in order to be powered (p < .05, 80% power, GPOWER3.1) to be able to detect the statistically medium to large sized effects of portion size on next day food intake and portion size norms as observed in (Robinson & Kersbergen, 2018), whilst accounting for any likely data exclusions from analyses. Participants were recruited from staff and students at the University of Liverpool, UK. Exclusion criteria were any history of food allergies, eating disorders and currently pregnant or following a weight loss/gain programme. Staff participated in exchange for a small monetary reimbursement and students participated in exchange for the same monetary reimbursement or course credit (1st year psychology students only). The study was approved by the University of Liverpool Research Ethics Committee.

### 2.3 Portion size conditions (session one)

Mikado chocolate covered biscuit sticks were the test food (manufacturer recommended serving size: 25 g which equates to approx. 11 sticks and 120 kcal). To determine portion sizes used, a pilot study was conducted in which female participants were shown images of portion sizes ranging from 2 to 24 sticks and asked which portion sizes were ‘small’, ‘normal’ and ‘large’. On the basis of this, during session one, in the ‘smaller portion size’ condition participants were provided with a serving of 6 biscuit sticks (14 g) and in the ‘larger portion size’ condition participants were shown a serving of 16 biscuit sticks (37 g), both on the same standard white side plate. Therefore, the difference in food volume relative to the manufacturers recommended serving size was matched between conditions.

### 2.4 Participant experience questionnaire (session two)

Participants reported on their age and gender, before completing filler items about their experience in the study. One item ‘I felt as though the amount of food I was eating would be measured by the researcher’ (5 point Likert scale response format, strongly disagree to strongly agree) was included to examine whether any effect of experimental condition on later food intake was dependent on the extent to which participants believed their food intake would be measured by the researcher (Robinson et al., 2014, Robinson et al., 2015). Participants were also asked ‘what do you think this study was attempting to find out’ (open ended) in order to exclude any participants from analyses that were aware of the study aims.

### 2.5 Portion size questionnaire (session two)

Participants were shown 12 images of portion sizes of the biscuit sticks (2 sticks to 24 sticks increasing in ascending order). To measure portion size normality participants were asked to select one in response to ‘Which of the following portion sizes would you say is a normal portion size of chocolate covered biscuit sticks to eat for a snack?’. As a manipulation check participants were shown the same scale again and then asked which portion size they had been served the previous day. Participants were also asked additional items on plate clearing habits and portion sizes that were not collected for the purpose of this study. The study questionnaires and study data are available in full at https://osf.io/m26qc/.

### 2.6 Procedure

As snack food was used, sessions took place during weekday mornings (10 am-12 pm) and afternoons (2 pm-4 pm). We did not formally attempt to standardise pre-session hunger, although participants were informed they may be required to eat snack food. After providing informed consent and confirming eligibility, participants were told by the researcher that the aim of the study was to test how mood, food and personality interact with one another. Participants first filled out a 10-item filler personality questionnaire to corroborate the cover story, as well as a series of 11 mood measures, including a measure of baseline hunger (‘how hungry are you right now’ 100 mm line scale, anchors: ‘not at all’ and ‘extremely’). Participants were then told that they would be provided with a serving of snack food to make a series of sensory ratings on and were left for 5 min to complete the ratings. The researcher explained that in the first session they were required to not eat the food and base their ratings on the appearance of the food. Participants completed a series of 100 mm line scales (anchors: ‘not at all’ and ‘extremely’) on sensory aspects of the food (e.g. ‘sweet’, ‘crunchy’) to corroborate the cover story (all participants followed the instruction of not eating). Participants then completed the same mood questionnaire again to further corroborate the cover story, before being scheduled to return at approximately the same time the following day. During the second session, participants completed a different set of 10 filler personality items and the same mood measures as in session one. The researcher then brought a large opaque plastic box that contained 33 biscuit sticks (the amount provided in a standard box of the product) and instructed participants that they were required to complete the same sensory ratings as in the first session after having tasted the food. The researcher then explained to the participant that she needed to print a final questionnaire and explained that they could serve themselves and eat as much as they wanted, leaving the same standard white plate on the table as used in session one. Five minutes later the researcher returned, took away the remaining food and participants completed a final battery of questionnaires that included (in order) a final set of mood measures to corroborate the cover story, the Dutch Eating Behaviour Questionnaire (Van Strien, Frijters, Bergers, & Defares, 1986), the participant experience and portion size questionnaires. Participants then had their weight and height measured by the researcher using a stadiometer and digital scales, before being debriefed.

### 2.7 Primary planned analysis strategy

We made an a-priori decision to exclude any participants from analyses who had directly guessed the aims of the study. We examined the effect of portion size condition on the amount of food participants ate the next day using an independent samples *t*-test. We compared the two portion size conditions on the portion size normality measure using a Mann-Whitney *U* test. To examine whether the effect of portion size condition on food consumption was explained by differences in portion size normality, we first examined whether portion size normality and food intake were correlated, before using the PROCESS macro version 3.2 for SPSS (Hayes, 2013). In order to test mediation we expressed the portion size normality measure (mediator) as a continuous measure (number of biscuit sticks). The independent variable was experimental condition (dichotomous data) and the dependent variable was grams consumed (continuous data). All analyses were conducted in SPSS 24.0.

## 3 Results

### 3.1 Participant characteristics

111 participants were recruited. Two participants correctly identified the aims of the experiment as relating to portion size (coded by two researchers) and were excluded from analyses, leaving a final sample size of 109 participants. See Table 1 for participant characteristics.

**Table 1:** Participant Characteristics.

	Larger portion size condition (n = 54)	Smaller portion size condition (n = 55)	Full sample
Age (years)	22.1 (4.9)	25.1 (8.4)	23.6 (7.0)
BMI (weight/height^2^)	23.2 (3.5)^a^	23.3 (4.1)^b^	23.2 (3.8)
Baseline hunger, session 1	41.5 (23.0)	37.5 (24.0)	39.5 (23.5)
Baseline hunger, session 2	36.6 (22.2)	42.0 (26.5)	39.3 (24.5)
DEQ, restrained eating	2.6 (0.9)^c^	2.6 (0.9)	2.6 (0.9)
DEQ, emotional eating	2.6 (0.8)^c^	2.7 (0.8)	2.7 (0.8)
DEQ, external eating	3.4 (0.5)^a^	3.5 (0.7)	3.4 (0.6)

Values are mean (SD).

DEBQ subscales are scored 1–5, with higher scores denoting greater restrained, emotional and external eating.

Due to missing data, n = ^a^52, ^b^51, ^c^53.

### 3.2 Portion size normality and food intake

In line with hypotheses, during session two participants believed a normal sized portion of the snack food to be significantly smaller if they had seen the smaller (N = 55) as opposed to larger (N = 54) portion size during session one. See Table 2. Moreover, participants exposed to the smaller portion size as opposed to the larger portion size during went on to consume significantly less snack food in session two. See Table 2. Although portion size normality and food intake were significantly correlated (Pearson’s r = 0.23, p = .018), the indirect effect of portion size condition affecting food intake via portion size normality was not statistically significant (Indirect effect = −0.89, SE = 0.70, BCa 95% CI = [−2.54, 0.15], proportion of total effect explained by indirect effect = 16.81%).

**Table 2:** Later food intake, portion size normality and manipulation check data.

	Larger portion size condition (n = 54)	Smaller portion size condition (n = 55)	Statistical comparison of portion size conditions
Food intake (grams)^a^	19.3 (14.2)	14.0 (11.0)	t(107) = 2.2, *d* = 0.42, p = .03 (independent samples *t*-test)
Portion size perceived normality^b^	8 (18)	6 (18)	U = 1125.5, z = −2.2, p = .025 (Mann Whitney-*U* test)
Portion size manipulation check^b^	14 (18)	8 (18)	U = 505.5, z = −6.0, p < .001 (Mann Whitney-*U* test)

a Denotes mean (SD) grams of biscuit sticks eaten in session two. Each biscuit stick weighs approximately 2.3 g.

b Denotes median (range) number of biscuit sticks selected from portion size questionnaire in response to items measuring perception of a normal sized portion and memory of portion size served, both measured in session two.

### 3.3 Sensitivity analyses

To examine whether the effect that portion size condition had on food intake was dependent on whether participants believed their food intake would be measured by the researcher, we examined the interaction (mean centred) between portion size condition and participant beliefs about whether their food intake would be measured in a forced entry linear regression model (entered alongside portion size condition and participant beliefs). The interaction term was non-significant (β = 0.06, p = .69), indicating that the effect of portion size condition on later snack food intake was not dependent on the extent to which participants believed the amount of food they ate would be measured. We also examined the effect of controlling for baseline hunger and participant characteristics (BMI, DEBQ scores) as covariates on the effect of condition on snack food consumption and the results remained the same as in the main analysis (p < .05).

### 3.4 Manipulation check

On average, participants in the smaller portion size condition tended to remember being served a significantly smaller portion than participants in the larger portion size condition. See Table 2. However, in the smaller portion size condition a substantial proportion of participants incorrectly recalled being served more than the amount they were served (29/55, 52.7%) and in the larger portion size condition a proportion incorrectly recalled being served less than they actually were (31/54, 57.4%).

## 4 General discussion

Participants exposed to a smaller as opposed to a larger portion of food subsequently believed that a ‘normal’ sized portion of that food was smaller and chose to eat less of that food a day later. These findings are consistent with the proposition that portion sizes can signal normative information and in doing so indirectly cause people to eat more or less of a food in future (Robinson & Kersbergen, 2018). Given that many food portion sizes have grown and this has coincided with the obesity crisis (Young & Nestle, 2002), the present work also provides some of the first direct evidence that the removal of supersized portions and reductions to the size of standard food products may not only limit the amount of food people consume from those products, but also change portion size preferences.

We hypothesised that the effect of portion size on later food intake would be explained by portion size altering perceptions of portion size normality. Although perceptions of portion size normality and later food intake were significantly correlated and differences in portion size normality accounted for 16% of the effect that portion size condition had on later food intake, our formal mediation analysis was not statistically significant. This may be because the effects that portion size had on later food intake and portion size normality were smaller than in previous research (Robinson & Kersbergen, 2018), which then resulted in reduced statistical power in the present study. Given that we used a different type of food in the present study (sweet snack food) and there were other methodological differences between these studies, it is difficult to ascertain why the effects observed in the present study were somewhat smaller in statistical size. Additionally, a number of participants incorrectly recalled the size of portion they had been visually exposed to and this may indicate that the lack of significant mediation effect was due to some participants not attending to the initial portion size they were exposed to. The only research we are aware of to have examined whether portion size alters future food preferences involved participants *consuming* small vs. large portions of a main meal food (Robinson & Kersbergen, 2018). The present study conceptually replicates those findings but suggests that mere visual exposure (as opposed to consuming smaller portions) can result in changes to portion size norms. A series of studies have also shown that exposure to images of small or large portion sizes of food affects portion size evaluations, but not immediate food intake (Robinson et al., 2016). The results of the present study may therefore suggest that a portion size of food needs to be physically present in one’s immediate food environment in order for portion size to communicate normative information that alters consumer behaviour.

The present study used a controlled experimental design to infer causality and the methods and analyses used also minimised the potential influence of demand characteristics on study findings (Robinson, Bevelander, Field, & Jones, 2018). Because this was a laboratory study in female participants only we cannot infer whether our findings would replicate in more realistic settings or in male participants, nor can we conclude whether our findings would be the same if we used a range of other foods or measured food intake beyond twenty-four hours. Although the test food used was palatable, is sold in mainstream UK supermarkets and previous data collected in our laboratory suggests that it is a food most participants would have been familiar with, in the present study we did not measure participant familiarity with the test food. Although participants were randomly assigned to conditions, not measuring participant familiarity is a limitation of the present study and measurement of this variable would have allowed us to examine whether the two experimental groups were similarly familiar with the test food used. Familiarity will therefore be important to consider in future research that builds on the initial evidence provided here. A further reason to examine the importance of familiarity is that familiarity with a food may moderate effects of portion size exposure. Longer lasting effects of portion size exposure on consumer preference may be most likely when the food is relatively unfamiliar (Robinson, Blissett, & Higgs, 2013) and/or when consumers have not developed learnt expectations about the satiety providing effects of a food (Brunstrom, Shakeshaft, & Scott-Samuel, 2008). Although a recent study found that eating a smaller portion size of a food affected later portion size preference (Robinson & Kersbergen, 2018), which is suggestive that the portion sizes of a food we are exposed to determine our preferred portion size of that food, irrespective of the satiety providing effects of the food.

We also reason that the effect that a smaller vs. larger portion size has on later eating behaviour may be likely to be specific to the food used, as consumers are likely to have distinct normative beliefs about portion sizes for different foods (Haynes et al., 2019), but this remains an empirical question. We manipulated portion size by varying the number of biscuit sticks (units) across conditions (as consumers would experience in the real world), therefore it may be the case that our results would be different if number of units was held constant and instead unit size was manipulated, although we reason this is unlikely because multiple studies have now shown that exposure to amorphous food portion sizes alters perceptions of portion size normality (Robinson and Kersbergen, 2018, Robinson et al., 2016). Although a no portion size exposure control condition may be a useful addition to future research, because portion size is on a relative visual continuum, providing that the small and large stimuli (portion size) that participants are exposed to are equidistant from participants’ existing ‘norm’ we presume that exposure to smaller and larger portion sizes would decrease and increase portion size norms respectively, as has been shown in other studies of food evaluations (Robinson et al., 2016).

## 5 Conclusions

Environmental exposure to smaller, as opposed to larger portion sizes of food may change perceptions of what constitutes a normal amount of food to eat and affect the amount of food people choose to eat in future.

## 6 Ethics approval and consent to participate

The study was approved by the University of Liverpool Research Ethics Committee and participants provided written informed consent.

## 7 Consent for publication

Not applicable.

## 8 Availability of data and materials

The materials and data set for this study are available at https://osf.io/m26qc/.

## Competing interests

ER has previously received research funding from the American Beverage Association and Unilever but does not consider this a conflict of interest.

## Funding

ER’s salary is supported by the MRC (MR/N000218/1).

## Author contributions

ER, JH and GK designed the study. JH and IK were responsible for data collection. ER and IK were responsible for data analysis. ER drafted the manuscript. All authors were responsible for the final approval of the manuscript and had full access to the data.
